# Youth Empowered Advocating for Health (YEAH): Facilitating Partnerships Between Prevention Scientists and Black Youth to Promote Health Equity

**DOI:** 10.1007/s11121-022-01450-9

**Published:** 2022-10-26

**Authors:** Briana Woods-Jaeger, Tasfia Jahangir, Devin Lucas, Marjorie Freeman, Tiffaney L. Renfro, Kristin E. Knutzen, Nkosi Cave, Melvin Jackson, Caroline Chandler, Christa Riggins, Alexandra F. Lightfoot

**Affiliations:** 1 Rollins School of Public Health, Emory University, Grace Crum Rollins Building1518 Clifton Road NE, Atlanta, GA #52630322 USA; 2 YEAH Community Consultant, Raleigh, NC USA; 3 Southeast Raleigh YMCA, Raleigh, NC USA; 4 Gillings School of Global Public Health, University of North Carolina at Chapel Hill, Chapel Hill, NC USA

**Keywords:** Structural racism, Photovoice, Participatory research, Black youth, Health equity

## Abstract

Structural racism inflicts a disproportionate burden of stress and trauma within Black communities, resulting in physical and mental health inequities that impact Black youth. Yet few multilevel interventions exist to address these deeply rooted inequities from a preventive standpoint, and even fewer are informed by the participatory input of the impacted communities. To bridge these gaps, we developed a community-based prevention strategy that promotes agency and active resistance to structural racism, Youth Empowered Advocating for Health (YEAH), and implemented it across various settings. We outline the development, implementation, and expansion of YEAH as a tool for promoting optimal health among Black communities. Lastly, we discuss lessons learned and offer a framework outlining key principles for prevention scientists to partner with Black youth and engage them in translational science to address structural racism. This framework is aimed at driving policies, practices, and procedures that promote equitable and sustainable change for and with Black communities.

## Background

Structural racism promotes discrimination through mutually-reinforcing systems (Bailey et al., 2017) and manifests as poverty, residential segregation, barriers to education and employment, and police and community violence. These experiences yield a disproportionate burden of stress and trauma within Black communities, resulting in physical and mental health inequities (Woods-Jaeger et al., 2020). Such health inequities among Black youth require prevention approaches informed by culturally responsive theory and research. The Radical Healing Framework (French et al., 2020) is a strengths-based model that promotes agency and active resistance to oppression as a means of fostering wellness at all levels of the social ecology. By centering justice and resistance against oppression as the vehicle for health equity, the framework calls for interventions that target multiple levels of the social ecology. Yet, few multilevel interventions exist to prevent health inequities informed by communities most impacted. Addressing this gap, we developed a community-based prevention strategy that promotes agency and active resistance to structural racism, Youth Empowered Advocating for Health (YEAH), and implemented it in various settings. We describe YEAH and how it has informed multilevel preventive intervention strategies that promote optimal health for Black communities. Additionally, we discuss lessons learned during the development and implementation of YEAH. These lessons inform our current research agenda examining YEAH as both a tool for research, and a strategy for intervention, to promote wellness among Black youth. Through sharing the development and implementation of YEAH, we offer a framework and key principles for how prevention scientists can partner with youth to engage in translational science that impacts policies, practices, and procedures to promote equitable and sustainable change for Black communities.

## YEAH: a Multi-level Prevention Strategy that Prioritizes Black Health

In partnership with communities impacted by stress and trauma-related health inequities, we have been implementing Youth Empowered Advocating for Health (YEAH) with Black youth in multiple states to identify multilevel intervention targets that promote optimal health for Black communities. YEAH follows the principles of youth participatory action research (YPAR), including equitably partnering with youth to better understand youth experiences, improve youth outcomes, and influence youth environments (Anyon et al., 2018; Kennedy et al., 2019). YPAR supports youth and adults working together to identify and address social problems (Anyon et al., 2018) by engaging youth in the process of (1) identifying intervention priorities and strategies (Lindquist-Grantz & Abraczinskas, 2020) and (2) participating in decision-making to mitigate inequities (Anyon et al., 2018; Kennedy et al., 2019). One systematic review of 63 studies found youth engagement in YPAR positively influenced agency and leadership (75.0%), academic or career (55.8%), social (36.5%), interpersonal (34.6%), and cognitive (23.1%) outcomes (Anyon et al., 2018). Another study found that 57.1% of YPAR studies report positive environmental outcomes, defined as “positive changes among adults, peers, organizations, and/or institutions” (Kennedy et al., 2019). Despite several of these studies focusing on minoritized youth and inequities in a broad range of settings, none explicitly addressed racial trauma or built on Black cultural assets to tackle structural racism. YEAH is a youth-adult developed curriculum that guides youth in identifying factors that influence health inequities in their community and addressing them through social action (Woods-Jaeger et al., 2013). The goals of YEAH are threefold: (1) to serve as an organizing tool for facilitating community-academic partnerships to promote health equity; (2) to promote youth as advocates for health equity by developing critical consciousness and skills in policy advocacy; and (3) to enable youth and adult community and academic partners to translate research to relevant social change through multilevel intervention development. Through its development and implementation over a decade, YEAH projects have consistently addressed topics related to structural racism, and over time we have observed how nurturing Black cultural assets has enhanced YEAH’s ability to promote tackling structural racism.

**Fig. 1:**
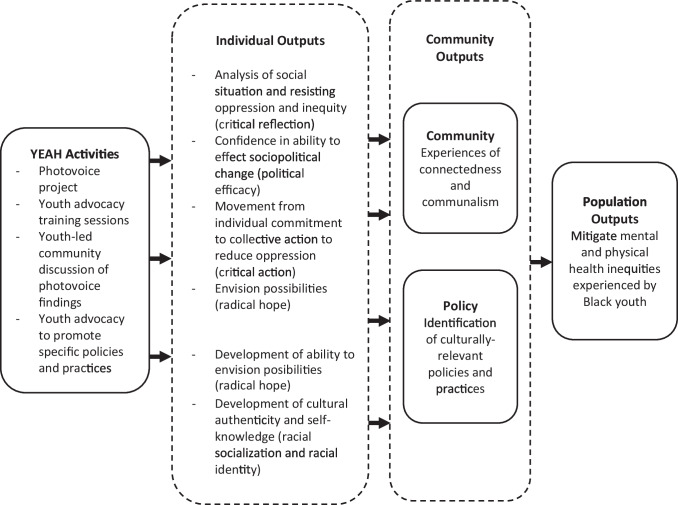
YEAH logic model

YEAH includes a photovoice project aimed at cultivating critical reflection, agency, and action to understand and address the root causes of social problems. The photovoice project is followed by advocacy training to support youth in pursuing their identified social action priorities. This youth-partnered research and action brings together multigenerational, multiracial coalitions of youth, university students, university faculty, community members, and key stakeholders, building on cultural assets to prevent health inequities. The logic model in Fig. 1 outlines the activities and outputs of YEAH. Table 1 list examples of deliverables borne out of the curriculum.

**Table 1:** YEAH curriculum and deliverables

Session #	Activity	Example deliverables from YEAH implementations
1–4	Photovoice project	Youth partner co-authored manuscripts and presentations; youth curated photo- exhibits
5	Planning and holding a community forum	Youth-led YEAH community forums (in-person or online) in Raleigh, KC, and Atlanta
6	Researching allies and past efforts	Invite lists for YEAH community forums that include key stakeholders that can enact change
6	Policy Solutions & Power mapping	Invite lists for YEAH community forums that include key stakeholders that can enact change
6	“How to ask”	Action Booths at YEAH community forums with specific asks for community to support
7	Community inventory	Proposed partnerships to pursue social action priorities
8	Communication styles	Action booths and open forum discussion led by youth during YEAH community forums
8	Using media for strategic communication	2022 “Do You See What We See” media Campaign
8	Create your own social media awareness campaigns	2022 “Do You See What We See” media campaign
8	Networking	Proposed partnerships to pursue social action priorities
8	Video advocacy and script-writing	2022 “Do You See What We See” media campaign
8	Creating a tip sheet to educate others	Challenging stereotypes handout provided at 2012 YEAH community forum
9	Partnering with existing organizations in the community	Continuation of YEAH without grant funding
9	Creating your own group	Continuation of YEAH without grant funding
9	Developing a mission statement	Continuation of YEAH without grant funding

## Origins of YEAH: North Carolina 10 Years of Implementation

YEAH emerged from a CBPR study that engaged Black youth (ages 12–17) and their parents in Raleigh, NC, in testing an adolescent HIV/AIDS prevention curriculum in faith-based settings. The study was initiated by Strengthening the Black Family, Inc. (STBF), a community-based organization in the historically Black community of Southeast Raleigh, which had sponsored a peer-led HIV education program for adolescents, Teens Against AIDS (TAA), for nearly 20 years. When the TAA funding ended, STBF, led by co-author Melvin Jackson, identified university-based funding and reached out to a researcher at the University of North Carolina at Chapel Hill, co-author Alexandra Lightfoot, to launch a study to build partnerships with local churches (Lightfoot et al., 2012; Woods-Jaeger et al., 2014) to test the HIV prevention curriculum, Focus on Youth + ImPACT, and propose adaptations to increase its relevance and appropriateness for faith settings (Lightfoot et al., 2014). Joined by then postdoc, lead author Briana Woods-Jaeger, we worked alongside a Community Advisory Board (CAB), comprised of diverse and intergenerational stakeholders, including faith leaders, educators, public health practitioners, parents, and youth members who actively participated in all aspects of the study to ensure relevance and appropriateness. Findings from focus groups with youth members of the CAB spurred our team to look beyond the behavior components addressed in the FOY curriculum towards the social and environmental factors that affect Black youth vulnerability to HIV. To do this, we conducted a photovoice project with 12 youth to examine their perceptions of what put them at risk for HIV and determine actions to intervene (Woods-Jaeger et al., 2013). For the photovoice sessions, the 12 youth were divided into two groups of 7 young men and 5 young women. Both groups followed the standard photovoice process but generated different photo assignments based on their evolving discussions. These audio-recorded and transcribed discussions were analyzed according to conventional content analysis procedures, using an inductive team coding approach. The final themes were member-checked with and endorsed by the youth. Our analysis of the photo discussions revealed how social inequities perpetuated by racism translated into HIV risk and highlighted several action steps to reduce HIV disparities. Specifically, youth participants discussed their experiences of being exposed to poverty, violence, and risky behaviors and its impacts on them and their peers. They described the limited opportunities within their physical environments—neighborhoods and schools alike—and barriers to their positive development within the social environment, where low expectations of Black youth became self-fulfilling prophecies for many of their peers (Woods-Jaeger et al., 2013). Within neighborhoods, the absence of opportunities elicited a sense of futility among youth which they conceptualized as “getting stuck.” Furthermore, they internalized incarceration and the cyclical nature of poverty as expected experiences in their lives. Lastly, while participants recognized social support as a protective factor against HIV risk behaviors, they attributed a lack of social support to the social inequities and the community-level resource deficiencies they faced (Woods-Jaeger et al., 2013). Participants also explained their socialization within their environments at the institutional, community, and interpersonal levels. At the institutional level, youth described the general lack of support or, as they observed, “lack of care for the Black community,” which was mirrored throughout their social and community interactions. At the school level, this included punitive approaches towards Black students with law enforcement officers, who often resorted to the use of force. At the community level, youth discussed internalizing from a young age many of the negative stereotypes about Black youth as sexually promiscuous, “ghetto,” and unlikely to succeed. At the interpersonal level, they were exposed to substantial sexual activities, distracting them from their studies and making them feel unsafe in school environments. Youth described how these combined exposures and experiences perpetuated feelings of hopelessness and a lack of agency among Black youth and, in turn, promoted HIV risk behaviors (Woods-Jaeger et al., 2013).

As a result of photovoice discussions, youth devised multiple action steps to address the social and structural factors they identified that affect Black youths’ vulnerability to HIV risk behaviors. Specifically, they advocated to (a) increase neighborhood opportunities and resources for adolescents, (b) increase institutional support in schools, churches, and community-based organizations, and (c) combat racial stereotypes. Supported by adult facilitators (co-authors of this manuscript), youth also conducted a forum to raise awareness, open conversation in their community, and galvanize action on the multi-level intervention priorities they identified. One key action pursued directly in response to these findings was the launch of YEAH as a mechanism for youth (with adult support) for engaging in ongoing advocacy to identify and address youth health inequities. A curriculum for YEAH was developed to build youth leadership, capacity, and advocacy skills (Woods-Jaeger et al., unpublished manual). YEAH aimed to increase institutional support through school and community outreach, tip sheets, and partnerships with schools, churches, and community-based organizations (CBOs). It expanded neighborhood opportunities by being implemented in neighborhood CBOs. Furthermore, it endeavored to combat racial stereotypes by raising awareness of the adverse consequences of such stereotypes through community forums and social media.

Since its inception, YEAH has continued to be implemented to promote youth-academic partnerships and address youth-identified concerns engaging 50 youth partners, 27 graduate student facilitators, 6 community partner leads, and 4 prevention scientists over time (Freeman et al., 2013; Rankins et al., 2014a, b; Riggins et al., 2018a; Woods-Jaeger et al., in press). Across implementations, a consistent theme has been addressing various aspects of structural racism. With YEAH as a guidepost, 25 additional youth in Southeast Raleigh have continued to engage in research and advocacy projects over the past 10 years. These projects have provided meaningful and sustained opportunities for youth to build their leadership and advocacy skills (Freeman et al., 2013; Rankins et al., 2014a, b; Riggins et al., 2018a). Their photovoice research has brought to light community concerns that affect youth, such as gentrification, structural inequality, the impacts of racism on youth mental health, and access to mental health support services (Riggins et al., 2019; Chandler et al., 2020a, b). Through asset mapping and photovoice, youth have identified the strengths of their community, as well as potential enhancements and translated this research to impact policies, practices, and procedures that promote equitable and sustainable change in their communities. Specifically, their work has informed the development of a purpose-built community, Southeast Raleigh Promise, which co-locates housing, education, wellness, economic opportunity, and leadership development to address systemic needs at the community level (Riggins et al., 2017, 2018b). In North Carolina, involvement in YEAH has provided a mechanism for youth to propose and contribute to solutions, including advocating for and helping to design a peer-led mental health curriculum, Honoring Our Power and Emotions (HOPE Seekers), a ten-week program now being used in Southeast Raleigh, aimed at supporting youth in developing individual mental health skills to navigate adolescence and beyond (Chandler et al., 2021).

## Expansion of YEAH: Kansas City and Atlanta Implementation

While YEAH has continued to evolve in NC, the approach has also been adapted and implemented in new locations, Kansas City and Atlanta. In response to the public health crises of community violence and racism, YEAH was implemented virtually with Black youth in Kansas City and Atlanta (ages 13–18) during the COVID-19 pandemic. The goal of this implementation was to engage youth exposed to community violence and racism in a critical analysis of multi-level contributors to these public health crises and identify social action priorities. Like previous implementations, identifying priorities to address structural racism remained a central focus. Fundamental to this implementation was developing strategies to maintain and build upon the integrity of the Critical Consciousness (CC) framework within the virtual environment. Specifically, we endeavored the promotion of (1) critical reflection, (2) political efficacy, and (3) social action, the CC framework’s three core components (Jemal, 2017). The 13 youth partners that participated in this implementation used photographs, drawings, and narratives from their lived experiences to critically reflect on community issues, build their sense of agency and political efficacy, and develop possible mechanisms of social action.

Youth Ambassadors, a community-based organization that utilizes a strengths-based approach to develop job and life skills and promote resilience among underserved youth (ages 14–18) and Boys and Girls Club of Metro Atlanta, a trusted national organization with local sites in Metro Atlanta intentionally focused on creating safe, inclusive, and engaging environments for youth, served as key community partners in this implementation of YEAH. Both partners identified the escalating community violence as a priority area for action and wanted to engage youth as part of the solution. These partners served as a bridge to build trust, credibility, and integrity in the process. They aided in recruitment and session facilitation efforts, enabling the critical engagement of youth in a virtual environment. Due to their knowledge of community resources and opportunities, our community partners were also vital in leveraging resources and opportunities to build youth agency, leadership capacity, advocacy skills, and political efficacy for social action.

Themes from photovoice in these locations highlighted youth experiences of historical racism, institutional racism, cultural racism, and internalized racism. Youth also discussed how these various manifestations of racism run deep through a long history that is passed down through families, institutions (i.e., schools), and collective narratives and how these burdens are endured without sufficient support from non-Black communities (Woods-Jaeger et al., in press). Youth emphasized the significance of protecting and preserving Black history, having pride in Black history, representation in education, responsibility of education for both oneself and others, descriptions of specific regional historical context, and generational knowledge. Youth posited that the promotion of Black history knowledge may reduce internalized, cultural, and institutional racism and effectively serve as a prevention strategy to reduce community violence (Woods-Jaeger et al., in press). In addition, youth discussed the importance of shifting focus away from stigmatizing the Black community regarding community violence and instead uplifting and showcasing community strengths such as mentoring relationships, as an anti-racist approach to community violence prevention. This has driven multi-level intervention community violence prevention priorities including (1) a community awareness campaign focused on increasing knowledge of positive Black history through a public service announcement, billboard campaign, and documentary style video with community testimonials; (2) institutional advocacy focused on promoting culturally responsive curriculum in schools; and (3) advocating for resources for mentorship programs to mitigate the intersecting effects of racism and community violence.

## Lessons Learned Through YEAH Implementation

Through the evolution of YEAH, we have learned that it is critical to be flexible and adapt to the resources and context of each local site while adhering to core principles. Guided by YPAR and CC principles, YEAH aims to build trust with adults and researchers as well as youth ownership of the research and action process. Youths’ creative and introspective capacity, along with their unique perspectives and lived experiences, are central to each session of YEAH. YEAH promotes youth decision-making opportunities throughout the process, including shaping photo assignments (Table 2) and submission formats, mediums, and modes of engagement, and active engagement in the entire research process, from qualitative theme development, to manuscript drafting, to conference presentations and social action planning, decision-making, and implementation based on research findings. Through these processes, we promote Critical Consciousness, defined as the ability to recognize oppressive factors in a community, understand the mechanism through which they work, and develop ideas to take action against them (Jemal, 2017). Further, opportunities for prevention scientists to partner with youth to engage in translational science that impacts policies, practices, and procedures are facilitated through YEAH and in our experience have resulted in sustainable changes for Black communities.

**Table 2:** Example recent photovoice assignments generated by youth across communities

	Kansas City	Atlanta	Raleigh
Assignment 1	How does living in fear affect how we see the world?	How can equality be spread through future youth among their peers?	Why does racism still live? Why are people racist?
Assignment 2	What do you think a perspective of equality would look like?	Can finding similarities between genders stop shootings and abuse?	How do we see/feel racial stereotypes in our day to day lives?
Assignment 3	How does cultural appropriation affect you and your community?	How can promoting good things shed light on issues in the community?	What brings races together versus what keeps them apart?

However, implementing YEAH has also come with a unique set of challenges in each setting. In its initial stages, YEAH was sponsored by Strengthening the Black Family, Inc., then a vital, longstanding non-profit with deep roots in the Southeast Raleigh community, working in collaboration with university-based partners. We had funding through STBF, co-author Melvin Jackson on staff, and a project coordinator dedicated to youth engagement and trusted by parents. This enabled us to continue to grow YEAH with multiple cohorts of youth to explore concerns important to them. We were also successful at raising small pots of funding from local sources to support this work over time. When STBF closed its doors in 2017, we were no longer able to support the project coordinator through this partnership. Melvin Jackson, however, took on a new role with Southeast Raleigh Promise which enabled us to continue YEAH with the involvement of graduate students from UNC’s Gillings School of Global Public Health. More recently, we have made another transition, now working closely with a community-based partner formerly at SERP who is now at The Resiliency Collaborative, who is our point person for youth engagement in YEAH. This evolution highlights both the potential and the cautions of this kind of work, as well as the value of having a strong and enduring partnership between the community and the university at its heart. With this partnership in place and ingenuity and flexibility, we are able to tap community (i.e., sources of funding, community-based expertise, trust among youth and parents) and university (i.e., sources of funding, committed faculty, new crops of graduate students, research expertise) resources and weather changes on the ground in the community to continue to support youth involvement in YPAR through YEAH.

When expanding YEAH to Kansas City and Atlanta, we had initial funding through a small pilot grant from the Robert Wood Johnson Foundation. However, this funding did not extend to cover the time and resources needed to implement the social action priorities developed through implementing YEAH in these two communities. In addition, we were challenged with overcoming the constraints created by the COVID-19 pandemic. During our initial sessions, youth reported a lack of familiarity with one another as well as the academic partners. To overcome this and promote trust-building, engagement, and youth ownership of space, we added additional components to our photovoice process. These included a mentorship model involving paired youth and research team members for check-ins and rapport-building between sessions and smaller informal group calls of three to five youth between sessions (Jahangir et al., in press). Additionally, lack of continued funding posed key challenges to pursuing social action priorities identified. To address these challenges, each partner leveraged existing resources and networks to support continued work on social action priorities and engaged in collaborative grant writing to secure additional resources.

Despite the challenges described above, we also witnessed the strengths of YEAH in different communities due to the commitment generated through academic-community partnership that goes beyond funded grants. We have observed how YEAH impacts the youth and adult partners who work towards social action that addresses social and structural determinants of health inequities experienced by Black youth. YEAH appears to promote key cultural assets among Black youth including racial identity and communalistic coping (Gaylord-Harden et al., 2012) through promoting collective resistance against racism at multiple levels with the support of academic and community adult mentors and partners. We recently qualitatively examined the process of racial identity development during our implementation of YEAH in Kansas City and Atlanta and identified increases in youth statements reflecting private racial regard (i.e., positive attitudes about Black Americans) and pride in racial history over the course of sessions (Cave et al., under review). Through the process of developing CC and positive racial identity, youth can identify particular manifestations of racism at multiple levels in their community, leverage cultural assets, and engage in collective resistance to prevent further harm by partnering with academic partners to translate research to action.

The social positionality and critical consciousness of the prevention scientist-community partner research team is a fundamental facilitator of YEAH’s success. All implementations of YEAH in Raleigh, Kansas City, and Atlanta were carried out by diverse teams, with each team member possessing their own unique identities. Black members, always the leads of the team, brought their lived experience of racial stressors and other forms of marginalization to the work. White members, as supportive advocates, brought critical understanding of the manifestations and impacts of structural racism and scrutiny of the role of whiteness in perpetuating inequity across our social systems. The common thread among all teams was a shared understanding of collective power—that healing and change are rarely products of individual attributes, and instead dependent on dynamic relational processes requiring supportive networks, communities, and structures.

Overall, our observations of YEAH implementation and youth engagement over the years lead us to believe that YEAH holds great promise as an intervention that addresses racial trauma. In particular, we posit that YEAH incorporates key tenets of the Radical Healing Framework, an important new framework that seeks to advance understanding of healing from racial trauma by focusing on collective, in addition to individual, approaches (French et al., 2020). Undergirding the framework is a call to interrogate the context of race and racism in the USA, and it promotes constructs essential to radical healing: critical consciousness, cultural authenticity and self-knowledge, emotional and social support, radical hope, and strength and resistance (French et al., 2020). Looking at YEAH through a Radical Healing lens provides the opportunity to think about how this work may offer a protective intervention strategy for Black youth. Our goal going forward is to systematically investigate YEAH’s mental health-promoting potential for Black youth exposed to racism and to identify lessons learned that can help prevention research re-envision policies, practices, and procedures to promote equitable and sustainable change for Black communities. Table 3 summarizes our lessons learned.

**Table 3:** Lessons learned from the implementation of YEAH

Lesson learned	Description
Lesson 1: YEAH facilitates trust-building between youth and academic researchers by promoting youth ownership of the research and action process To promote youth ownership, it is critical that prevention scientists recognize, give space, and honor youth as experts in their own lived experiences	YPAR and CC principles guide the YEAH process. YEAH promotes youth decision-making opportunities throughout the process, including shaping photo submission formats, mediums, and modes of engagement; active engagement in the entire research process, from qualitative theme development, to manuscript drafting, to conference presentations and social action planning, decision-making, and implementation based on research findings. It is recommended that prevention scientists form diverse teams and invest the time and resources to form equitable youth partnerships that sustain over time
Lesson 2: YEAH promotes partnerships between youth and prevention scientists to engage in translational science. To translate science into sustainable change, it is critical that prevention scientists contribute to building collective power (e.g., networks, resources, and structures) that last beyond a specific grant	YEAH facilitates youth engagement in translational science by promoting partnerships between academic and youth partners to engage in research that impacts policies, practices, and procedures to promote sustainable changes in Black communities. Through the process of developing CC youth and prevention, scientists have identified manifestations of racism at multiple levels in communities and translated research to action. It is recommended that prevention scientists and community partners proactively plan for sustained engagement in social action planning that extends beyond current funding and the tenure of current research and youth partners
Lesson 3: YEAH holds promise as an intervention for racial trauma. To better understand processes that prevent harms from racism, it is critical that prevention scientists study the work of Black scientists who have led the way in racial trauma scholarship	YEAH incorporates key tenets of the Radical Healing Framework (French et al., 2020) and appears to promote cultural assets among Black youth including racial identity, racial socialization, and communalistic coping (Gaylord-Harden et al., 2012). It is recommended that prevention scientists examine how processes promoted through YEAH may offer a preventive intervention strategy for Black youth exposed to racial trauma to mitigate associated physical and mental health harms

## Future Research Questions

Informed by the Radical Healing Framework, as well as observations of how cultural assets like racial identity, racial socialization, and culturally-relevant coping are nurtured during the implementation of YEAH, one of the primary research questions we have identified regarding YEAH is to better understand its impact on the youth who participate—particularly, how it may nurture Black cultural assets to promote radical healing from racism. Gaylord-Harden and colleagues (Gaylord-Harden et al., 2012) offer a culturally situated, asset-based framework for Black youth exposed to racial discrimination that highlights the buffering effects of three cultural assets: racial socialization, racial identity, and culturally relevant coping. In implementing YEAH, we have observed processes of racial socialization through youth-adult relationship building and youth racial identity development and expression, as well as culturally-relevant coping, namely, through promoting communalism and connectedness. Examining these processes and how they influence outcomes for youth participating in YEAH are priority research questions for our team.

## Does YEAH Promote Black Racial Identity?

Black racial identity has emerged as a salient theme in our implementations of YEAH, leading to our recent assessment of how racial identity develops over the course of YEAH (Cave et al., 2022). Black racial identity comprises one’s private regard (i.e., positive feelings towards one’s racial group) and public regard (i.e., the extent to which others view the Black race positively). Youth development of racial identity can affect their wellbeing throughout adolescence and into adulthood (Marks et al., 2020). Positive views of one’s own racial identity are associated with mental, physical, and psychological health (Marks et al., 2020). Further, positive racial identity has been shown to be protective against racism-related stress and adverse mental health outcomes (Spencer et al., 1997; Volpe et al., 2019) including depression (Hardeman et al., 2016) and trauma (Wright et al., 2017). Assessing if and how YEAH promotes racial identity is a priority area of future research.

## How Do Adult Facilitators and Peers in YEAH Engage in Racial Socialization?

It appears through our 10 years of implementing YEAH that racial socialization is occurring throughout YEAH as facilitators and peers engage in critical reflection and dialogue about racism. Racial socialization includes messaging from parents and other adults that instill pride in youths’ racial/ethnic group membership (Coard & Sellers, 2005). This cultural asset can also provide Black adolescents with strategies for coping with racial discrimination (Coard & Sellers, 2005; Richardson et al., 2015), improve psychological adjustment, (Neblett et al., 2008), and decrease depressive symptoms (Davis & Stevenson, 2006). Additionally, racial socialization influences the development of racial identity as youth enter adolescence (Neblett et al., 2009). Better understanding how this occurs and the contribution of adult facilitators and peers within YEAH is an area of great interest.

## Does YEAH Promote Culturally Relevant Coping?

Over our decade of implementing YEAH, we have observed that YEAH nurtures culturally relevant coping. For Black Americans, culturally relevant coping may be grounded in historical, cultural, and philosophical traditions of people of African descent, representing an African-centered (or Africentric) worldview. Culturally relevant coping based on an Africentric worldview is described as communalistic coping (Gaylord-Harden et al., 2012). The cultural value of communalism emphasizes the importance of Black individuals’ shared responsibility for each other and their community (Constantine et al., 2006) and embraces a “holistic system of support” to respond to racism (Jones, 2007). Communalistic coping has been associated with increased resilience among Black youth exposed to trauma and adversity (Constantine et al., 2006) and shown to buffer the relationship between racism-related stress and depressive symptoms (Chapman-Hilliard & Adams-Bass, 2016; Neblett et al., 2010). Relatedly, connectedness, or the nature of one’s relationship with society and feelings of support experienced in one’s community (Kim et al., 2015) is an important culturally relevant coping source. Positive feelings of connectedness contribute to resiliency and are protective against depression (Kim et al., 2015). YEAH engages youth in active research that informs social action and collective resistance in communities, thus promoting communalistic coping and potentially improving overall connectedness. Evaluating whether this relates to improved youth outcomes is an important future direction.

## Conclusion

As Black youth continue to navigate contexts of racism-related stress and trauma, it is critical to foster safe and generative spaces for healing and resistance. Incorporating key aspects of the Radical Healing Framework (French et al., 2020), YEAH has great potential to serve not only as a facilitating structure for translational research, but also as an intervention that promotes health and wellbeing among Black youth. It is time for more prevention scientists to partner with Black youth to prevent the numerous negative physical and mental health outcomes associated with structural racism (e.g., depression, anxiety, suicidal ideation, asthma, obesity; Woods-Jaeger et al., 2020) through translational science to impact policies, practices, and procedures that promote health equity. In order to do this work, it is critical that prevention scientists first invest time in building equitable relationships with both youth and adult community partners. In building these relationships, prevention scientists should work with community partners to establish research questions and identify participatory methods for collecting and analyzing data, disseminating findings, and holding each other accountable to YPAR principles. Prevention scientists must also be intentional in creating spaces where youth are given latitude to be leaders in this work and recognize youth as experts in their own lived experiences. YEAH has the potential to both facilitate the development of these type of partnerships and translational research, as well as promote Black youth wellness in the process through nurturing cultural assets and promoting Radical Healing from racial trauma. Future research warrants an examination of the multi-level outcomes of YEAH (e.g., individual mental and physical health, interpersonal relationships and social networks, community and institution-level change in policies, practices, and procedures). As we move into our second decade of implementing YEAH, we look forward to examining these research questions and continuing to build and nurture partnerships between prevention scientists and Black youth to promote Radical Healing and health equity.
