# Correction: Youth Empowered Advocating for Health (YEAH): Facilitating Partnerships Between Prevention Scientists and Black Youth to Promote Health Equity

**DOI:** 10.1007/s11121-024-01686-7

**Published:** 2024-05-24

**Authors:** Briana Woods-Jaeger, Tasfia Jahangir, Devin Lucas, Marjorie Freeman, Tiffaney L. Renfro, Kristin E. Knutzen, Nkosi Cave, Melvin Jackson, Caroline Chandler, Christa Riggins, Alexandra F. Lightfoot

**Affiliations:** 1 Rollins School of Public Health, Emory University, Grace Crum Rollins Building 1518 Clifton Road NE, Atlanta, GA #52630322 USA; 2 YEAH Community Consultant, Raleigh, NC USA; 3 Southeast Raleigh YMCA, Raleigh, NC USA; 4 Gillings School of Global Public Health, University of North Carolina at Chapel Hill, Chapel Hill, NC USA

**Correction to: Prevention Science (2022) 25:20–30** 10.1007/s11121-022-01450-9

The article “Youth Empowered Advocating for Health (YEAH): Facilitating Partnerships Between Prevention Scientists and Black Youth to Promote Health Equity”, written by Woods-Jaeger, B., Jahangir, T., Lucas, D., Freeman, M., Renfro, T.L., Knutzen, K.E., Cave, N., Jackson, M., Chandler, C., Riggins, C., and Lightfoot, A.F., was originally published Online First without Open Access. After publication in volume 25, issue 1, page 20–30 the author decided to opt for Open Choice and to make the article an Open Access publication. Therefore, the copyright of the article has been changed to © The Author(s) 2022 and the article is forthwith distributed under the terms of the Creative Commons Attribution 4.0 International License, which permits use, sharing, adaptation, distribution and reproduction in any medium or format, as long as you give appropriate credit to the original author(s) and the source, provide a link to the Creative Commons licence, and indicate if changes were made. The images or other third party material in this article are included in the article’s Creative Commons licence, unless indicated otherwise in a credit line to the material. If material is not included in the article’s Creative Commons licence and your intended use is not permitted by statutory regulation or exceeds the permitted use, you will need to obtain permission directly from the copyright holder. To view a copy of this licence, visit http://creativecommons.org/licenses/by/4.0/.

The original article has been corrected.

